# Fast tracking the design of theory-based KT interventions through a consensus process

**DOI:** 10.1186/s13012-015-0213-5

**Published:** 2015-02-11

**Authors:** André E Bussières, Fadi Al Zoubi, Jeffrey A Quon, Sara Ahmed, Aliki Thomas, Kent Stuber, Sandy Sajko, Simon French, Members of the Canadian Chiropractic Guideline Initiative

**Affiliations:** School of Physical and Occupational Therapy, Faculty of Medicine, McGill University, 3630 Promenade Sir-William-Osler, H3G 1Y5, Montreal, QC Canada; Département chiropratique, Université du Québec à Trois-Rivières, Trois-Rivières, QC Canada; Centre de recherche interdisciplinaire en réadaptation (CRIR), Montréal, QC Canada; School of Population and Public Health, Faculty of Medicine, University of British Columbia, Vancouver, BC Canada; International Collaboration on Repair Discoveries (ICORD), Vancouver Coastal Health Research Institute, Vancouver, BC Canada; Spine Program, Department of Orthopaedics, Faculty of Medicine, University of British Columbia, Vancouver, BC Canada; Clinical Epidemiology, McGill University Health Center, Montréal, QC Canada; Center for Medical Education, Faculty of Medicine, McGill University, Montréal, QC Canada; Division of Graduate Education and Research, Canadian Memorial Chiropractic College, Toronto, ON Canada; Private practice, Toronto, ON Canada; School of Rehabilitation Therapy, Faculty of Health Sciences, Queen’s University, Kingston, ON Canada

**Keywords:** Theoretical domains framework, Knowledge translation, Interviews, Content analysis, Intervention design, Multifaceted intervention, Chiropractic, Neck pain, Self-management

## Abstract

**Background:**

Despite available evidence for optimal management of spinal pain, poor adherence to guidelines and wide variations in healthcare services persist. One of the objectives of the Canadian Chiropractic Guideline Initiative is to develop and evaluate targeted theory- and evidence-informed interventions to improve the management of non-specific neck pain by chiropractors. In order to systematically develop a knowledge translation (KT) intervention underpinned by the Theoretical Domains Framework (TDF), we explored the factors perceived to influence the use of multimodal care to manage non-specific neck pain, and mapped behaviour change techniques to key theoretical domains.

**Methods:**

Individual telephone interviews exploring beliefs about managing neck pain were conducted with a purposive sample of 13 chiropractors. The interview guide was based upon the TDF. Interviews were digitally recorded, transcribed verbatim and analysed by two independent assessors using thematic content analysis. A 15-member expert panel formally met to design a KT intervention.

**Results:**

Nine TDF domains were identified as likely relevant. Key beliefs (*and relevant domains of the TDF*) included the following: influence of formal training, colleagues and patients on clinicians (*Social Influences*); availability of educational material (*Environmental Context and Resources*); and better clinical outcomes reinforcing the use of multimodal care (*Reinforcement*). Facilitating factors considered important included better communication (*Skills*); audits of patients’ treatment-related outcomes (*Behavioural Regulation*); awareness and agreement with guidelines (*Knowledge*); and tailoring of multimodal care (*Memory, Attention and Decision Processes*). Clinicians conveyed conflicting beliefs about perceived threats to professional autonomy (*Social/Professional Role and Identity*) and speed of recovery from either applying or ignoring the practice recommendations (*Beliefs about Consequences*). The expert panel mapped behaviour change techniques to key theoretical domains and identified relevant KT strategies and modes of delivery to increase the use of multimodal care among chiropractors.

**Conclusions:**

A multifaceted KT educational intervention targeting chiropractors’ management of neck pain was developed. The KT intervention consisted of an online education webinar series, clinical vignettes and a video underpinned by the Brief Action Planning model. The intervention was designed to reflect key theoretical domains, behaviour change techniques and intervention components. The effectiveness of the proposed intervention remains to be tested.

**Electronic supplementary material:**

The online version of this article (doi:10.1186/s13012-015-0213-5) contains supplementary material, which is available to authorized users.

## Background

In 2010, neck pain was identified as one of the leading causes of years lived with disability (YLDs) [1,2]. The estimated 1-year incidence of neck pain ranges between 10% and 21% with a higher incidence noted in office and computer workers [3]. While between 33% and 65% of people have recovered from an episode of neck pain at 1 year, most cases run an episodic course over a person’s lifetime, and thus, relapses are common [3]. In chiropractic practice, neck pain accounts for approximately 25% of initial consultations [4].

Recently, the Canadian Chiropractic Association (CCA) and the Canadian Federation of Chiropractic Regulatory and Educational Accrediting Boards (CFCREAB) updated a Clinical Practice Guideline (CPG) on the management of non-specific neck pain to help improve the delivery of chiropractic care for patients with this condition [5]. The guideline recommends that either a noninvasive combined approach or multimodal protocol including manual therapy, advice about self-management, and physical activity including exercise is an effective treatment strategy for acute and chronic neck pain. The promotion of physical activity, including exercise, is considered paramount to the prevention and treatment of musculoskeletal pain and related co-morbidities [6]. Concomitant advice about self-management is also important [7]. Despite these recommendations, a recent survey of Canadian chiropractors suggests that only 41% of respondents provide advice to patients on self-management strategies [8]. Furthermore, a survey of chronic neck and back pain patients indicated that less than half of attending healthcare practitioners, including physicians, chiropractors and physical therapists, prescribed exercise [9].

Poor implementation of scientific evidence into clinical practice has important population health ramifications including delayed recovery and increased disability levels and costs [10,11]. The design and evaluation of theory-based complex interventions (i.e. interventions involving many interacting components) are increasingly recommended for studies aiming to implement evidence into practice [12-14]. While a recent overview of systematic reviews offered no compelling evidence that multifaceted interventions are more effective than single-component interventions [15], the extent to which behavioural interventions were theory-based was not specified in this overview. Further, there is currently no generally accepted method of categorizing elements of an intervention, so misclassification may have occurred. Multi-component interventions may be more effective than single interventions if they simultaneously target determinants of behaviour change [16]. Systematic methods that incorporate both an understanding of the determinants of practice behaviours and a scheme for categorizing elements of knowledge translation (KT) interventions in relation to those determinants were recently proposed [17]. As the implementation of guidelines requires targeting clinicians to change their behaviour, it may be helpful to base implementation strategies on explanatory frameworks explicitly concerned with behaviour change [18,19]. Psychosocial theories have been successfully applied to explore potential modifiable determinants of behaviour change [20-25] and to help design targeted interventions to change clinical practice [17,26,27]. The Theoretical Domains Framework (TDF) was developed expressly for this purpose [28] and has been used by researchers across several healthcare systems to identify implementation barriers and inform corresponding interventions [29]. A refined version of the original TDF is available for use by researchers [30]. Few published studies have explored the usefulness of theoretical constructs specifically among chiropractors [17,24].

### Context and purpose of the study

This project is part of an ongoing effort to facilitate the implementation of clinical guidelines and best practices into chiropractic settings in Canada [31]. The Canadian Chiropractic Guideline Initiative (CCGI) was launched by both the CCA and the CFCREAB over a decade ago. The overall goal of the initiative is to improve chiropractic care delivery in Canada through the development, dissemination and implementation of CPGs.

The aim of the current project was to design a KT intervention to facilitate the uptake of a recently developed guideline for the management of non-specific neck pain among chiropractors [5].

### Study objectives

Specific objectives were to 1) identify chiropractors’ beliefs about managing non-specific neck pain through multimodal care and to explore barriers and facilitators to implementing evidence-based management recommendations for neck pain, and 2) map behaviour change techniques to key TDF domains and to develop a KT intervention for chiropractors to support the use of the neck pain guideline.

### Ethical approval

Approval for this project was granted by the ethics review boards of both the Canadian Memorial Chiropractic College in Toronto, Canada (1310X07) and McGill University in Montreal, Canada (A11-B55-13B).

## Methods

**Figure 1:**
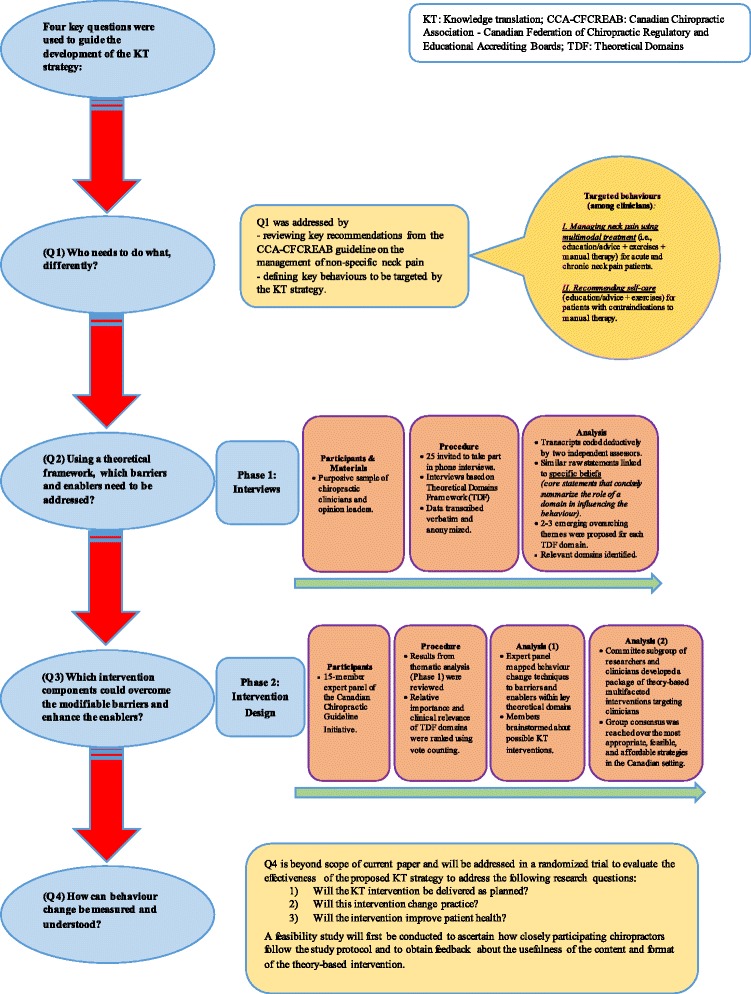
**Flowchart—KT intervention design.**

We developed a KT intervention using a systematic, theoretically informed approach (Figure 1). The TDF was utilized to identify chiropractors’ beliefs and barriers and enablers of behavioural change (objective 1) and to subsequently choose theory-informed behaviour change interventions to implement evidence into practice (objective 2) [17]. We used an approach for developing KT interventions that applies the answers to four key questions to guide the development of the strategy: (1) Who needs to do what differently? (2) Which modifiable barriers and enablers need to be addressed? (3) Which interventions could overcome those barriers and enhance the enablers? and (4) How can behaviour change be measured and understood?

The first key question “Who needs to do what, differently?” was addressed by specifying the target behaviours required to implement the neck pain guideline into practice. This was achieved by reviewing the key recommendations from the updated CCA-CFCREAB guideline on the management of non-specific neck pain [5], which specifically stated that chiropractic practitioners needed to do the following: “Manage neck pain using *multimodal treatment* (i.e. education/advice + exercises + manual therapy) for acute and chronic neck pain patients” and “Recommend *self-care* (i.e. education/advice + exercises) for patients with and without contraindications to manual therapy”. These two recommended modalities (multimodal treatment and self-care) were chosen because they have been demonstrated in high-quality evidence to be beneficial for spinal pain patients with early or persistent symptoms [32,33].

Subsequently, key questions (2) and (3) were addressed in separate phases (phase 1—interviews and phase 2—intervention design).

Key question (4) “How can behaviour change be measured and understood?” is beyond the scope of the current paper. However, relevant future steps are included in the “Discussion” section.

### Phase 1—interviews

This phase addressed key question (2): Using the TDF, which barriers and enablers need to be addressed?

#### Participants

Participants were chiropractors in full-time practice and/or had prolonged experience as educators or leaders within professional chiropractic organizations. A purposive sample of 13 chiropractors was drawn from the CCA membership list (*N* = 7,200) to capture respondents representing a broad range of geographical areas, years in practice and expertise (field practice as well as decision-makers). Members of the CCGI were asked to recommend and agree on potential respondents who were likely to fulfil these criteria.

## Material

An interview topic guide based on the TDF was developed [28,30] (see Additional file 1). Questions were informed by previous publications on the topic [34-40]. Probing questions were used where necessary for further clarification [41]. Face and content validity of the interview guide was initially assessed by experts in KT (AB, SF, SA, AT) and chiropractic practice (KS, SS, JQ). The number of questions ranged from 1 to 4 for each of 13 TDF domains, for a total of 28 questions.

### Procedure

In November 2013, an original purposive sample of 25 practitioners was invited to participate in telephone interviews. The first 13 respondents were followed up by email and telephone to confirm participation. Prior to the interviews, participants completed a consent form and reviewed relevant recommendations of the Neck Pain Guideline. Interviews lasted 60 min and were audio recorded. Field notes were taken concurrently. Data were transcribed verbatim and anonymized prior to analysis. The initial findings were then reviewed by the 15-member committee attending a 2-day meeting to identify barriers and enablers.

### Phase 2—intervention design

This phase addressed key question (3): Which intervention components could overcome the modifiable barriers and enhance the enablers?

#### Participants

Fifteen members of the CCGI attended a 2-day meeting to design a complex KT intervention targeting clinicians. Committee members included seven researchers including four KT experts familiar with behaviour change interventions, three graduate students, two faculty members (one each from the Canadian Memorial Chiropractic College and the Université du Québec à Trois-Rivières), two chiropractic private practitioners and one communications specialist. The remainder of this paper focuses on the process used to fast track the identification of important component interventions and the design of a complex KT intervention for clinicians.

#### Procedure

Results from the telephone interviews and the classification of statements into domains were reviewed by the 15 committee members. A subgroup, composed of researchers (AB, JQ, SF, AT, FAZ) and clinicians (KS, SS), then met to systematically develop a clinician-centred package of theory-based interventions. Team members were first asked to consider the main findings from phase 1. Members were also invited to review the definitions of the TDF domains [30] and an article on evidence-supported behaviour change techniques [42] before proceeding with intervention mapping. Based on published systematic reviews of the literature on the effectiveness of various interventions [43-45] and feasibility considerations, committee members then considered the mode of delivery for the KT intervention.

## Analysis

### Phase 1—identification of barriers and enablers

Transcripts obtained during phase 1 were coded deductively by two independent assessors, reviewed by the principle investigator (AEB) and submitted for comments to three investigators (KS, AT, SF). Disagreements were formally resolved by consensus at each step. Consensus was defined as agreement among at least three out of the four investigators. The analysis plan was similar to the one used by team members in previous qualitative studies among chiropractors [24,25], adapting a method proposed by colleagues [40,46], and further refined in a series of papers recently published in *Implementation Science* [29]. Each statement was initially coded into relevant TDF domains onto an Excel spreadsheet. Coding was guided by our understanding of the domain definitions and of the constructs within a domain. Statement responses were then linked with *specific beliefs*. A specific belief is defined as a core statement that captures a common theme from multiple response statements and provides detail about the role of a given domain in influencing practice behaviour [40]. Within each domain, emerging overarching themes were identified. An example of the coding process is provided in the “Results” section under the domain of Social Influences.

To assess whether or not we achieved data saturation [47], we conducted concurrent data analysis and coding. Themes started to recur by the third interview, and no new themes emerged after the 11th interview. To ensure a robust and defensible coding of the data into beliefs and relevant domains, a health psychologist with TDF expertise later critiqued our analysis and interrogated the coding of domains considered potentially problematic by meeting CCGI attendees.

The initial findings were reviewed by the 15-member committee attending a 2-day meeting. In groups of three, participants scrutinized up to four different domains and resolved any additional coding disagreements through discussion. Specific beliefs and overarching themes were summarized by two investigators (AB, JQ) and again reviewed for disagreement by all meeting participants. Finally, domain relevance was determined independently by investigators based on three criteria: 1) presence of conflicting component beliefs; 2) evidence of strong component beliefs that were perceived to impact practitioner behaviour; and 3) a high frequency of specific beliefs [39,48], each of which was weighted equally to judge a domain’s likelihood of influencing the targeted behaviours.

### Phase 2—identification of intervention components

For phase 2, the TDF domains deemed relevant in phase 1 were individually ranked by meeting participants (from most to least relevant based on criteria mentioned above). Key TDF domains were identified based on perceived clinical relevance and vote counting. Domains with few vote counts were formally discussed and either included or excluded based on consensus. Two researchers (AB, SF) mapped key domains to behaviour change techniques (BCTs) [49]. Meeting attendees then brainstormed about possible effective KT interventions utilizing these BCTs and also determined modes of delivery and actions. Committee members then reached consensus over the modes of delivery and possible actions based on feasibility and likely costs. A transcriptionist noted the conclusions of discussions for each step. The final decision about the KT interventions, modes of delivery and actions was made by study investigators based on best available evidence [43-45].

## Results

### Phase 1—interviews

#### Characteristics of participants

Telephone interviews were conducted with nine chiropractors and four leaders/decision-makers in the profession within six Canadian provinces. The average age of interview participants was 46.7 years (SD ± 6.8); 30% (4/13) were females, and the average number of years in practice was 20.8 years (SD ± 7.2). These sample characteristics were consistent with national averages for Canadian chiropractors (Table 1).

**Table 1:** **Subject characteristics**

**Characteristics (% unless stated otherwise)**	**Participants (** ***N*** **= 13)**	**CCRD (2011)** ^a^ **(** ***N*** **= 2,296)**
Professional background	Chiropractors (*n* = 9)	N/A
	Chiropractic leaders/decision-makers (*n* = 4)	
Age	46.7 years (SD ± 6.8)	44.3 years (SD ± 11.7)
Gender	70% M; 30% F	67.2% M; 32.8% F
Number of years in practice (mean and SD)	20.8 years (SD ± 7.2)	17 years (SD ± 11.8)
Type of practice	3 in solo practice	34.8% in solo practice
Patients presenting with complaints of neck pain	Over 40%	N/A
Prescribe exercise as a component of spinal pain treatments	100%	69.4% (SD ± 29.7)
Provide exercise leaflets to patients	69%	N/A
Has onsite low-technology exercise equipment	62%	N/A

*N/A* not available, *M* male, *F* female, *SD* standard deviation.

^a^Canadian Chiropractic Research Databank, maintained by the Canadian Chiropractic Association.

#### Key themes identified within relevant domains

We identified 450 statements representing 39 specific beliefs and 20 themes. Nine key domains were considered to have a greater influence on the targeted behaviour: 1) Social Influences; 2) Environmental Context and Resources; 3) Reinforcement; 4) Skills; 5) Behavioural Regulation; 6) Knowledge; 7) Memory, Attention and Decision Processes; 8) Social/Professional Role and Identity; and 9) Beliefs about Consequences (see Additional file 2). Four other domains (Beliefs about Capability, Intention, Goals, and Emotion) were considered to have a lesser influence on the targeted behaviour. Additional file 3 shows the specific beliefs and relevant domains together with illustrative quotes from our data.

### Phase 2—intervention design

Additional file 4 shows how different BCTs were mapped to key relevant domains and proposed KT interventions and actions. Based on published systematic reviews of the literature [43,44] and feasibility considerations, committee members agreed on the modes of delivery for the KT intervention.

### Domain-specific themes (phase 1) and proposed BCTs (phase 2)

In this section, we present the main responses and corresponding themes that underpinned each key relevant theoretical domain (from phase 1), followed by the BCTs and corresponding descriptions and rationales for the proposed interventions that were mapped to each domain (phase 2).

### Social Influences

#### Phase 1

Forty-eight statements were concerned with interpersonal processes that can cause individuals to change their thoughts, feelings or practice behaviours. Peer (20/48) and patient (19/48) approval was perceived as important in influencing chiropractors’ clinical decisions. For instance, participants said they often referred difficult cases to, and sought advice or second opinions from, colleagues. Many participants also reported that patient reactions would influence their management decisions. Two participants indicated that they would rather consult guidelines than their peers. Half of participants expressed little regard for the opinions of colleagues.

The following example provides insight into the coding process. For this domain, Social Influences, participants were asked two questions, one of which was, “Are there instances where you may consider consulting other people for their opinion regarding the need for providing proper patient education/advice and home exercises?” One practitioner’s response to this question was, “Yes, I believe in co-management with a personal trainer or physiotherapist.” This response was linked to the *specific belief* that *seeking advice from colleagues, referring difficult cases to colleagues or seeking a second opinion is important/not important* (see Additional file 3). The corresponding theme for this belief was *influence of others’ opinions* (e.g. colleagues, patients, organizations and new literature) (Additional file 2).

#### Phase 2

BCTs that were mapped onto the Social Influences domain included *social processes of encouragement*, *pressure* and *support*, and *modelling/demonstration of behaviour by others*. For our planned intervention, these BCTs will be delivered by opinion leaders through a series of webinars and short videos. This strategy is supported by a majority of interview participants who reported (in 39/48 statements) being influenced by peers when it comes to using new evidence. Opinion leaders (OLs) are people who are seen as likeable, trustworthy and influential and, therefore, may be able to persuade chiropractors to implement up-to-date evidence, make better decisions and optimize patient care [50,51].

### Environmental Context and Resources

#### Phase 1

Fifty-nine statements cited personal or environmental circumstances that either discouraged or encouraged the development of skills and abilities, independence, social competence or adaptive behaviour. Nearly all (12/13) participants indicated that they experienced no important environmental constraints when implementing multimodal care. However, in many statements (44/59), participants expressed a desire for additional exercise equipment or educational resources. Statements from four participants indicated that running behind schedule might affect their use of multimodal treatments on any particular day.

#### Phase 2

BCTs identified to facilitate the target behaviour consisted of *resources to facilitate the use of multimodal care*, including the following: 1) chart note templates with integrated multimodal treatment decision boxes to replace existing data collection forms in the office [52]; 2) sheets/handouts to facilitate the prescribing of exercises and other activities during routine appointments; 3) pocket cards as easy reference sources to reinforce important day-to-day clinical decision criteria [53]; and 4) an existing smartphone application for patients (Stanford Patient Education Research Center: http://patienteducation.stanford.edu/) to encourage them to increase their level of physical activity. We plan to provide public access to these tools on a new website of the CCGI (http://www.chiroguidelines.org), which already contains treatment information targeting practitioners, patients and decision-makers in the profession. Providing easy access to evidence-based information on ways to promote exercise and physical activity supports the key recommendations (multimodal care and self-care) and is an integral component of the proposed multifaceted KT intervention.

### Reinforcement and Skills

Although the TDF domains of *Reinforcement* and *Skills* are distinct, several behaviour change techniques targeting these domains share similar constructs. Furthermore, interventions proposed during the brainstorming session were applicable to both domains. Consequently, phase 1 and 2 results for both of these domains are combined here.

#### Phase 1

Eighteen participants indicated that better clinical outcomes would reinforce the use of multimodal treatments for neck pain patients. Of 30 statements concerning skills or proficiency acquired through practice, 25 referred to the importance of good doctor-patient communication skills for effectively managing neck pain patients using multimodal care. A few statements referred to the need for counselling skills (3/30) and to the importance of manual and technical skills (2/30).

#### Phase 2

A range of BCTs targeting reinforcement and skills were proposed, including *persuasive communication*, *graded tasks*, *increasing skills*, *motivational interviewing*, *social processes of encouragement*, *pressure*, *support*, *self-monitoring* and *demonstration of desired behaviours by others.* Persuasive doctor-patient communication skills are particularly relevant for empowering patients and raising their confidence to self-manage their health conditions and to adopt healthy behaviours. The use of a short video (as a dynamic visual presentation) to demonstrate desired behaviours may help reinforce the use of persuasive communication skills and teach novel strategies to encourage greater physical activity and a healthier lifestyle [44]. Several approaches from the behavioural change literature, such as Motivational Interviewing (MI) [54], the 5 A’s (Assess, Advise, Agree, Assist, Arrange) [55,56] and chronic disease self-management programmes [57] are potentially effective guides for clinicians and patients. Brief Action Planning (BAP) is a structured, stepped-care self-management support technique for chronic illness care and disease prevention and, because of its concise approach, is considered ideal for promoting behaviour change during individual clinical visits. BAP integrates the principles and practice of MI with goal setting and action planning concepts from the self-management support, self-efficacy and behaviour change literature [58]. Comprised of a series of three questions and five skills, the overall goal of BAP is to assist individuals to create an action plan for a self-management behaviour that they feel confident about achieving (reviewed in detail elsewhere [58]). BAP is currently being used in diverse care settings, including primary care, for the self-management of chronic illnesses and disabilities such as diabetes, depression, spinal cord injury, arthritis and hypertension. BAP is also being used to assist patients to develop action plans for disease prevention. A webinar to raise awareness on BAP and a short video illustrating how to apply BAP in the clinical setting will be developed by the Guideline Initiative and offered to Canadian chiropractors.

### Behavioural Regulation

#### Phase 1

A total of 53 statements cited factors aimed at managing or changing objectively observed or measured actions. Interview participants acknowledged the following strategies as being important for improving patient outcomes: regularly monitoring the patient’s condition, assessing the patient’s motivation to comply with advice and home exercise, encouraging exercise, providing multimodal care and adapting treatment plans to patients’ needs.

#### Phase 2

BCTs relevant to this domain included the use of *monitoring*, performance *contracts*, behaviour schedule *planning/implementation*, *prompts/triggers/cues*, and *use of imagery* or *mental rehearsal*. A majority of statements expressed the tendency of interview participants to monitor or objectively measure patient health outcomes. Others, however, admitted they neither routinely advise on nor monitor patients’ lifestyle changes. Therefore, examples of easy-to-use outcome behaviour monitoring strategies [49] will be incorporated into an educational webinar. Clinicians will be encouraged to think about their successes in implementing multimodal care and to subsequently document those experiences within easy-to-use paper or electronic checklists. The checklists, in turn, will be designed to constructively supplement or replace clinicians’ usual office visit forms.

### Knowledge

#### Phase 1

About half of respondents’ statements (28/59) expressed agreement with the recommendations of the neck pain guidelines, and nine additional statements suggested these guidelines fairly represented the evidence. Some participants suggested they lacked knowledge about exercise (6/59). The responses generally reflected confidence in the rigour of the guideline development process. Very few raised concerns about the operational definition of spinal manipulative therapy (SMT) used in the guideline; however, some respondents were disappointed by the lack of available research to either support the broader utilization of SMT or to inform specific dosage patterns.

#### Phase 2

*Information regarding the behaviour of interest* is pertinent considering that about one third of statements suggested that clinicians mostly rely on their experience (*n* = 9) or, alternatively, felt they lacked knowledge on exercise prescription (*n* = 6). Misconceptions about evidence-based practice (EBP) and the role of CPGs in clinical decision-making remain a substantial issue for some chiropractors [59,60], limiting their appreciation for the potential benefits of adhering to CPG recommendations. A better understanding of chiropractors’ clinical experiences and of the dissonance between their beliefs and research evidence may help translate research into practice and improve patient care [61]. We are therefore developing user-friendly evidence summaries in combination with active learning strategies, including videos and clinical vignettes embedded within an interactive webinar. These interventions will be aimed at improving clinicians’ understanding of EBP, CPGs, guideline recommendations and practitioner self-efficacy regarding the adoption of patient-centred behavioural health practices [62]. To make the webinars more interactive and engaging, a number of polls and quizzes will be included, along with pauses to allow attendees to reflect on their own practice behaviour. A question-and-answer period will be used at the end of each webinar.

### Memory, Attention and Decision Processes

#### Phase 1

Forty-three statements referred to the ability of participants to retain information, focus selectively on aspects of the practice environment and/or choose between two or more treatment alternatives. Twenty statements suggested that the participants were not challenged by decision-making, with some participants indicating that their practice was already in line with guideline recommendations. A few statements (5/43) suggested that some clinicians did not rely on algorithms to make a decision. Thirteen statements acknowledged the importance of considering patients’ psychological factors when deciding whether or not to recommend self-management.

#### Phase 2

Techniques mapped to this domain included *self-monitoring*, *planning/implementation*, *prompts/triggers/cues* and *motivational interviewing.* To encourage the use of multimodal treatment, clinicians will be provided with easy-to-use documentation and recordkeeping forms. Educational handouts, exercise prescriptions, pre-composed notes and referrals to other healthcare providers (especially for patients with psychological overlay) may further encourage clinicians to implement multimodal care.

### Social/Professional Role and Identity

#### Phase 1

Twenty-one statements related to a *coherent set of behaviours* and *personal qualities* of chiropractors within their work environment. While 16 relevant responses affirmed that it was congruent with the role of the chiropractor to employ multimodal care, other respondents felt differently, suggesting conflicting beliefs for this domain.

#### Phase 2

BCTs relevant to this domain included *social process of encouragement*, *pressure* and *support*. Three-quarters of the statements indicated that chiropractors believed that managing patients with neck pain using multimodal treatment is part of their role. However, other respondents felt their role was limited to providing monotherapy (SMT exclusively). Similar to the domain of Social Influences, these BCTs will be addressed by the delivery of the webinar series and short videos by opinion leaders.

### Beliefs about Consequences

#### Phase 1

Sixty-four statements related to the perceived consequences of managing patients with/without multimodal care*.* Twenty-two statements highlighted the likely benefits of using multimodal care, such as increased patient compliance and empowerment through self-care and better health outcomes (shorter recovery times, decreased pain, decreased headaches, better sleep). Conversely, 18 additional statements suggested that managing neck pain patients without using multimodal care would likely result in poor patient treatment response. Twenty-four additional statements did not appear to influence the target behaviours.

#### Phase 2

BCTs relevant to this domain included *persuasive communication*, *information regarding behaviour outcome*, *feedback* and *self-monitoring*. Overall, 40/64 statements appeared to support the use of multimodal care to improve patient health outcomes.

Both *persuasive communication* and *feedback* techniques will be incorporated into a webinar series informing practitioners of the purpose of evidence-informed practice and CPGs. Key messages such as “Using guidelines improves patient health”, “Better outcomes leads to increased referrals” and “Using multimodal care won’t slow down your practice” will be included into our presentations. Additionally, clinical vignettes will be developed to illustrate how to provide multimodal care, and a short video will demonstrate how to apply the BAP in the clinical setting to help patients set goals, adopt a healthier lifestyle and increase physical activity. *Self-monitoring* techniques may include standardized office recordkeeping forms that encourage documentation of prescribed evidence-based multimodal treatment.

### Multifaceted KT intervention

Committee members considered the multiple BCTs identified above and combined them into a deliverable intervention. Based on best available evidence [43-45] and feasibility considerations, committee members agreed that the mode of delivery for the KT intervention for clinicians would include four elements designed to reflect key theoretical domains and behaviour change techniques:

I. A 60-min platform presentation on the content of the CPG at continuing education events
II. Three 60-min webinars containing information on the following:
  - What evidence-informed practice is, and why CPGs are useful
  - Key CPG recommendations on the management of non-specific neck pain and
  - Self-management support strategies with a focus on the BAP model as an example
III. Two online case vignettes with problem-based decision-making exercises revolving around realistic scenarios
IV. A learning module with segmented videos demonstrating a clinician applying the BAP model to facilitate behaviour change (about exercise and other self-management) in a patient with chronic neck pain.

### Key question (4): How can behaviour change be measured and understood?

We are planning a pilot study for a cluster randomized trial to evaluate the feasibility of implementing the developed KT strategy into chiropractic clinical practice. This feasibility study will help to determine if the complex intervention and study protocol can be carried out as planned, and what will be the best outcomes for or means of measuring the effect of the intervention.

## Discussion

To our knowledge, this is the first study to explore chiropractors’ beliefs and attitudes about using a multimodal approach to manage non-specific neck pain, and then to use these findings to develop a KT intervention. Results from individual interviews among chiropractors conducted from six Canadian provinces highlighted potential barriers and facilitators to implementing a newly developed neck pain guideline. Adherence to prescribing multimodal care is influenced by many factors such as *Social Influences*; *Environmental Context and Resources*; *Reinforcement*; *Skills*; *Behavioural Regulation*; *Knowledge*; *Memory*, *Attention and Decision Processes*; *Social/Professional Role and Identity*; and *Beliefs about Consequences.* An expert panel mapped BCTs to barriers and enablers within key theoretical domains and identified relevant KT strategies and modes of delivery to increase the likelihood of use of multimodal care among chiropractors. Our findings provide the foundation to proceed with an implementation study, similar to an ongoing trial on the management of low back pain among Australian chiropractors [25].

Interview participants felt that better availability of and access to educational materials and pamphlets would encourage their implementation of the new guideline. To date, the beneficial effect of educational materials alone on clinical practice has been observed to be small [63]. However, even educational materials as a stand-alone intervention can shift practitioner beliefs, which in turn is an important precursor to shifting practitioner behaviours toward compliance with CPG recommendations for musculoskeletal disorders [64]. Furthermore, our findings are consistent with those of other studies demonstrating that across different professions, clinicians are more receptive to evidence from peers, textbooks and consensus proceedings than evidence from CPGs to inform them of recommended practice guidelines [65-70]. We expect that the use of opinion leaders and knowledge champions to convey key messages during our proposed webinars will greatly enhance the otherwise modest effect of practitioner-centred educational materials alone [50,71].

While participants were generally aware of and agreed with the guideline content (*Knowledge*), some admitted they preferred relying on their personal experience to guide treatment. These findings are consistent with other studies exploring clinicians’ beliefs toward research findings [72-74], in which regard, doctors of chiropractic (DCs) acknowledged that research is important but felt it was disconnected from daily clinical practice. Confidence in applying research into clinical practice also varied among our participants, some of whom cited lack of education and/or experience in EBP, critical appraisal and research skills as important obstacles to implementing research findings. Although undergraduate students at most chiropractic teaching institutions currently undertake formal coursework and training in research methods [75], our participants—who were representative of the national membership—had been in practice for a mean duration of 21 years and therefore predated the era of evidence-based practice in chiropractic curricula. Nonetheless, a comprehensive EBP curriculum has been shown to enhance measures of knowledge acquisition, skills for interpreting the scientific literature and prevalence of self-reported behaviours favouring the use of quality online resources by students [76]. We anticipate that an interactive webinar on the benefits of basing clinical decisions on evidence-informed practice and CPGs should yield similar benefits among practicing clinicians.

Most of our interview participants agreed that communication and technical skills are the key to effective patient management and good patient outcomes. Communication skills between healthcare providers and their patients are considered the “cornerstone” of patient education and are an important component of clinical management [77]. Patients generally value advice from their healthcare providers and feel that it effectively influences their health status [78]. Furthermore, well-designed patient education and self-management interventions have been found to significantly improve health outcomes for several conditions [77]. The common thread between different self-management approaches that strive to provide more effective guidance for clinicians and their patients is the use of strategies that facilitate collaborative decision-making, problem-solving and goal setting [54-57]. While these approaches have been widely endorsed [79-82] as a unifying framework for behavioural counselling in primary care [83-86], the selection of the best approach will depend on the clinical context [87,88]. For instance, different variables can affect the success of social communication and patient education, including practice characteristics (weekly patient practice volume), type of practice (solo versus group) and sex of practitioner [89]. These factors should be considered in the design of KT strategies to improve communication skills. Based on the principles and practice of motivational interviewing, self-efficacy and behaviour change literature, the BAP approach may be ideal for facilitating behaviour change (e.g. increases in physical activity) among patients with chronic conditions such as neck pain during routine clinical encounters [58].

Recent studies support the use of a combination of evidence from CPGs, clinicians’ expertise and patients’ preferences to inform clinical decisions [66,70,90]. The majority of our respondents indicated that the decision to undertake multimodal care was not particularly difficult (*Memory, Attention and Decision Processes* domain). However, none explicitly used decision algorithms, and few systematically relied on CPGs to inform the decision to use multimodal care. This is particularly relevant for complex cases of neck pain for which the use of manual care alone is often less effective than multimodal therapy incorporating a behavioural or psychosocial component [5,33].

Our interview participants expressed high intentions of managing neck pain patients in a way consistent with the guideline and were confident in their ability to manage neck pain (*Beliefs about Capabilities*). In other studies, health professionals often report strong intention, perceived behavioural control, positive attitudes and a strong normative influence [91,92], yet gaps continue to exist between available scientific evidence and customary clinical practice. A review of cognitive factors associated with the prediction of healthcare professionals’ intentions and behaviours suggests that about half of the unique variance explaining behavioural intention translates into professional behaviour [92]. Clinicians’ behaviours often require deliberate decision-making in complex contexts. For example, in behaviours such as providing self-management advice and chronic disease-related education, *automatic process* appears to be overridden by a more reflective process [93]. In such cases, decision-making likely involves both reflective (motivational and volitional) and automatic processes. Thus, interventions targeting both reflective processes (e.g. providing information, changing outcome expectations and setting goals) and automatic processes (e.g. contingent prompts/cues and rewards) may be more effective behaviour change strategies [93]. Specifically, interventions that incorporate BCTs aimed at translating behavioural intention into *actual* behaviour while focusing on post-motivational elements have greater potential to reduce evidence-practice gaps for people with chronic conditions [94-98].

Consistent with the operant learning theory (that consequences following a behaviour determine the probability that it will be repeated in the future) [99,100], a majority of participants felt that better patient outcomes with multimodal treatment would likely reinforce their adoption of this approach. Nonetheless, few clinicians indicated that they routinely measure patient progress with validated tools. This finding is in line with other evidence that the majority of DCs do not utilize objective measures to monitor the responses of their neck pain patients to care [101].

### Strengths and limitations

This study, which used a rapid interview process, face-to-face meetings with a large panel of researchers and clinicians, and scrutiny of findings by a behavioural psychologist, has provided valuable insight into the factors that may influence the use of multimodal care among chiropractors. Nonetheless, there were several limitations to our study, including the small number of participants and, therefore, potentially biased nature of our sample. However, we observed that similar themes started to recur by the third interview, and no new themes emerged after the 11th participant. We therefore believe that it is unlikely that the inclusion of more participants would have changed the overall balance between different beliefs favouring, versus those opposing, our targeted behaviours. On the other hand, we acknowledge that self-reported confidence in managing neck pain was quite high among our participants, and this may have reflected a form of volunteer bias. Meanwhile, 13 participants is typically an appropriate sample size for this type of qualitative study [47].

It is likely that other important barriers to guideline implementation would have surfaced had we also interviewed patients. Patient’s views and expectations may considerably influence clinicians’ practices and can be a barrier to the appropriate use of guidelines [102].

The domain of *Optimism*, not included in the original TDF [28], was not accounted for in our topic guide questionnaire, largely because the research team lacked clear guidance on how best to operationalize this construct. While our coding did not reveal specific beliefs that were a natural fit for this domain, it was felt that constructs related to this domain would be partly captured in other domains such as *Emotion* and *Social/Professional Role and Identity*.

Despite using a revised version of the TDF [30], challenges were encountered owing to the lack of clear definitions for *goals*, which rendered the achievement of consensus about the significance of this domain elusive at times. On the other hand, interview participants generally considered themselves as having already achieved the specific *goal* of implementing the guidelines. A recent exploratory study demonstrated the utility of goal facilitation and goal conflict for predicting the self-reported provision of physical activity advice by primary care health professionals (beyond intention and perceived behavioural control from the theory of planned behaviour) [103]. These findings suggest that different attributes within the goals domain clearly influence whether health professionals engage in guideline-recommended behaviours, and should therefore likely be considered within interventions aimed at increasing physical activity levels in patients.

While we have shown that the approach we used to develop this KT intervention [17] is feasible, the effectiveness of this approach has not yet been validated empirically. We therefore plan to evaluate the effectiveness of the KT intervention in a future study. In addition, professional experience and knowledge of theoretical concepts about the TDF varied greatly among our research team during phase 2 (the intervention design phase) of this project. This may have affected the ranking of key domains and the choice of corresponding interventions and modes of delivery. On the other hand, the engagement of KT experts familiar with behaviour change interventions, along with the inclusion of both practitioners and decision-makers in this development process, should enhance the credibility of our KT strategy among our target audiences. The impact of group composition among KT developers and implementers on both processes of care and patient outcomes also remains untested. Taking a rigorous approach to intervention design such as we have described can be resource intensive. However, the process can now be replicated more efficiently using the CCGI committee members to address other knowledge-practice gaps relevant to the scope of chiropractic practice.

## Conclusions

Few studies have attempted to examine potential barriers and facilitators to implementing guidelines among chiropractors using the TDF. Our study provides new insight into the beliefs and intentions of chiropractors contemplating multimodal management of neck pain patients, as well as the theory-based determinants of guideline compliance. Adherence to neck pain guideline recommendations appears to be influenced by a number of barriers and facilitators. Relevant TDF domains included *Social Influences*; *Environmental Context and Resources*; *Reinforcement*; *Skills*; *Behavioural Regulation*; *Knowledge*; *Memory*, *Attention and Decision Processes*; *Social/Professional Role and Identity*; and *Beliefs about Consequences.* These domains appeared to be important in light of their association with either a high frequency of relevant beliefs, presence of conflicting beliefs and/or evidence of strong beliefs likely to impact targeted behaviours. Our study findings informed the development of a theory-based KT intervention aimed at increasing the likelihood of clinicians to provide multimodal care for neck pain patients.

## Additional files

Additional file 1:
**Interview topic guide for individual interviews.**
Additional file 2:
**Thematic content analysis based on the TDF.**
Additional file 3:
**Findings of relevant domains together with illustrative quotes.**
Additional file 4:
**Mapping behaviour change techniques on key domains, proposed KT interventions and actions.**

## Abbreviations

BAP: Brief Action Planning
CCGI: Canadian Chiropractic Guideline Initiative
CMCC: Canadian Memorial Chiropractic College
CoP: Communities of practice
CPGs: Clinical practice guidelines
DCs: Doctors of chiropractic
EBP: Evidence-based practice
EHRs: Electronic health records
KT: Knowledge translation
TDF: Theoretical domain framework
