# Supplementary material for: Fast tracking the design of theory-based KT interventions through a consensus process
Source: Implement Sci. 2015 Feb 11;10:18. doi: 10.1186/s13012-015-0213-5 (PMC4330935; doi:10.1186/s13012-015-0213-5)
Supplement: Additional file 1: — Interview topic guide for individual interviews. [file 13012_2015_213_MOESM1_ESM.pdf]

Additional File 1. Interview topic guide for individual interviews

"Identifying factors likely to influence the use of guideline recommendations for the management of adult neck pain among Canadian chiropractors"

<u>Interview Guide</u>

I'd like to begin by asking you to state your name slowly for the benefit of the person who will be transcribing the interview.

Could you describe *how you manage new patients with neck pain in your office. By that I mean your usual routine once the patient is with you in the examining room, (such as case history questions), examination procedures, and whether managing acute or chronic neck pain patients using a multimodal treatment strategy (combining different therapies) is part of the initial visit. (Prompts: What do you feel you do well? What do you feel you could do better?)*

Thank you.

Now for the rest of the interview, I have some slightly more specific questions. Some may seem repetitive, but please bear with me. We would like to understand how chiropractors make practice decisions about managing patients with neck pain. I may also seek clarification during the interview via probing questions such as: *'What do you mean'; 'Would you explain that'; 'What were you thinking at the time'; 'Walk me through your experience', 'What skills do you need?'; 'How and why do you use it?' I would like to encourage you to say as much as you like, be as detailed and descriptive as you can.*

***** For the purpose of this interview, 'non-specific' refers to patients with uncomplicated mechanical neck pain that varies with time and activity with no neurologic deficits, fractures or indicators of potentially serious pathologies (i.e. red flags)***

The key recommendations from the Neck Pain Guidelines are:

1. Recommend multimodal treatment (education/advice + exercises + manual therapy) for acute and chronic neck pain patients;

2. Recommend self-care (education/advice + exercises) for patients with contraindications (either physical and/or psychological) to manual therapy.

****** For the purpose of this interview:***

1) ***Multimodal treatment is defined as a treatment plan that incorporates several possible interventions, in this case referring to education/advice, exercises and manual therapy.***

2) ***'Manual therapy' refers to the use of spinal manipulative therapy or chiropractic adjustment (high velocity, low amplitude thrust) and/or mobilisation (low velocity, high amplitude).***

Knowledge

1. *Tell me about the evidence surrounding optimal patient management using a multimodal treatment strategy for non-specific neck pain?*
   (Prompt: Did reading the guidelines improve your understanding?)
2. *Do you adhere to any specific guideline to help you make informed decisions about when to recommend a multimodal treatment plan, including specific advice and home exercises, to patients with neck pain?*
   (Prompts: How do you use it? Why do you use it? What do you think of it?
3. *Do you agree with the guideline contents?*
   (Prompt: What alternative, if any, would help you conform to the guideline's recommendations?)
4. *Are the guidelines representative of the evidence (quality, appropriateness)?*
   (Do the guidelines reflect the evidence?)

Beliefs about capabilities (self-efficacy)

5. *How confident are you in managing both acute and chronic non-specific neck pain using a multimodal treatment strategy?*
   (Prompt: How about education/advice? Exercises? Manual therapy?),
   (Prompt: How easy or difficult is this? What skills are required to manage patients with these conditions? What problems or barriers have you encountered in managing patients with neck pain?)

Behavioural regulation

1. *Do you monitor changes in patients' neck pain? Health status? After treating them using multimodal treatments? What is the usual outcome (do patients improve/deteriorate?)?*
2. *What could help you manage neck pain patients using a multimodal treatment strategy (i.e., education/advice + exercises + manual therapy)?*
3. *Do you assess the patient's motivation to follow advice and perform home exercises? If so, how? How do you manage a non-compliant patient?)*

Skills

4. *What new skills would you require to be able to manage neck pain patients using a multimodal treatment strategy?*
   (Prompts: additional training, counselling skills /communication techniques, continuing education, educational material, online information.)
5. *What do you think about the importance of communication skills for the management of patients using a multimodal treatment strategy? (Prompt: Why?)*
6. *What do you usually say or how do you manage patients who ask only to receive SMT/chiropractic adjustment but where you find it is not to their benefit?*

Intention

7. *On a scale where zero means 'none of the time' and five means 'most of the time', do you intend to manage non-specific neck pain patients using not only chiropractic adjustments but also education/advice and specific exercises?*

Goals

8. *What is the importance (i.e. priority) of education/advice + exercises in the context of other tasks (such as delivering SMT) in achieving the desired patient outcome?*
   (Prompt: Why do you feel that way?)

Memory, Attention and Decision Processes

9. *How easy or difficult is it to decide if a particular new patient needs a multimodal treatment plan or not?*
10. *What rules of thumb, if any, do you use to reach a decision?*
    (Prompts: decision rules, guidelines…)?

Reinforcement

11. *On a scale where zero means 'none of the time' and five means 'most of the time'', would you manage neck pain patients using a multimodal treatment strategy if the rewards were greater than when using manual therapy alone? (e.g., better patient satisfaction, less patient discomfort, etc.)*

Beliefs about consequences (Anticipated outcome/attitude)

12. *What are the benefits of managing a patient using a multimodal treatment strategy for acute and chronic non-specific neck pain?*
    (Prompt: better patient health outcomes? Shorter recovery times?)
13. *What are the potential disadvantages in managing patients with non-specific neck pain without offering proper advice or prescribing specific exercises?*
    (Prompts: 1) longer recovery time 2) patient preference and satisfaction)
14. *How do you perceive the safety of manual therapy, particularly with regards to cervical spine manipulation? Have these guidelines changed your impressions?*

Environmental context and resources (environmental constraints)

15. *Are there any factors in your practice likely to either help or prevent you from prescribing specific exercises to patients with non-specific neck pain?*
    (Prompts: Do you think having or not having onsite rehabilitation equipment (low tech or high tech) influences your decision to manage non-specific neck pain patients using a multimodal treatment strategy?
    Does running late or having a staff shortage on any particular day influence whether or not you decide to manage non-specific neck pain patients using a multimodal treatment strategy?
    Are there any resources available that you use to help you manage non-specific neck and back pain patients using a multimodal treatment strategy (information pamphlets or posters to inform patients about the benefits of regular exercise or specific neck exercises)?

Social influences (Norms)

16. *Are there instances where you may consider consulting other people for their opinion regarding the need for providing proper patient education/advice and home exercises? (Prompts: peers, managers, other professional groups, patients)*
17. *How do the views of other colleagues or organizations influence your decision to offer multimodal treatment to your patients?*

Emotion (Stress/concerns)

18. *How do you respond to distressed patients?*
19. *Is seeing acute neck patients in apparent distress likely to influence your decision to prescribe home exercise?*
20. *Does seeing a patient with chronic neck pain, particularly if there is a psychological overlay such as depression, influence your decision in terms of how you manage that patient with respect to home care or advice?*

Social/professional role and identity (self-standards)

21. *Do you think it is appropriate that your role should include managing patients with neck pain using a multimodal treatment strategy?*

Those are all of the questions I have for you today. Has anything else occurred to you about this topic that we haven't asked about? Are there any other thoughts you wish to convey on this topic?
