# Supplementary material for: Fast tracking the design of theory-based KT interventions through a consensus process
Source: Implement Sci. 2015 Feb 11;10:18. doi: 10.1186/s13012-015-0213-5 (PMC4330935; doi:10.1186/s13012-015-0213-5)
Supplement: Additional file 2: — Thematic content analysis based on the TDF. [file 13012_2015_213_MOESM2_ESM.pdf]

Additional File 2. Thematic content analysis based on the TDF

TDF domain†	Questions (N)	Utterances (N)	Specific beliefs (N)	Specific beliefs (number of utterances)	Increase[a] N (%)	Decrease[b] N (%)	No Influence N (%)	Themes
Social Influences	2	48	4	- I consult others during difficult cases (N=6).<br>-Colleagues, professional organizations, MDs, patients influence (14)/don't influence (5) my decision to use multimodal treatments (N=19)<br>- New guidelines may influence my decision to manage neck pain patients using multimodal care (N=2)<br>- Patients in apparent distress influences (19)/does not influence (2) my decision to prescribe home exercise (in terms of timing) (N=21)	39 (81)	2 (4)	7 (15)	Influence of others' opinion (colleagues, patients, organizations and new literature)
Environmental Context and Resources	3	59	4	- Printed (or electronic) Educational Material on exercise would help deliver multimodal treatments (N=33)<br>- Running late does (N=4)/does not influence (N=9) using multimodal treatments (N=13)<br>-Insurance coverage can be a barrier (N=2)<br>- A pamphlet about mechanical neck pain in general would be helpful (N=11)	44 (75)	6 (10)	9 (15)	Environmental resources;<br>patients tools within the clinic
Reinforcement	1	18	1	-Better clinical outcomes would reinforce the use of multimodal treatments for neck pain patients (N=18).	18 (100)	0	0	Better outcomes reinforce compliance
Skills	3	30	3	- Communication skills are the key for effective patient management and good patient outcomes: Persuasion (video, scripts, role play) (N=25)<br>- Technical & communication skills are important (N=2)<br>- I don't feel I need additional skills (N=3)	27 (90)	0	3 (10)	Clinician-Patient communication skill
Behavioural Regulation	3	53	4	- I monitor changes in patient's condition subjectively or using pain and/or functional scales. Outcomes are good (N=17)<br>- I assess (8)/ do not assess (3) patient motivation (subjectively or using a scale 1-10) to comply (N=11)<br>- Patient rarely ask for just SMT (N=8)<br>-Multimodal treatment is beneficial to everyone (N=9)	40 (75)	13 (25)	0	Assessing readiness for change<br><br>Post intentional behaviour

				- I do (6)/ do not know how to (2) adapt my treatment plan according to patient needs and the time I have (N=8)				
Knowledge	4	59	6	- I agree (28)/ partially disagree (3) with the content of the guideline (N=31)<br>- The guidelines are representative of the evidence (N=9)<br>- I don't use the guidelines directly but I already use multimodal treatments (N=2)<br>- The guideline offers no advice on diagnosis (N=2)<br>-I don't use the guideline. I rely on my clinical experience (N=9)<br>- I lack knowledge about best exercises (N=6)	37 (63)	22 (37)	0	Awareness of the guidelines; Knowledge of evidence
Memory, Attention and Decision Process	2	43	3	- Deciding if a new patient will receive multimodal treatment is easy (N=20)<br>- I use (5)/ I don't (5) use any rules of thumb. Decisions are based on individual cases (N=10)<br>- Patients with psychological overlay influence my decision to recommend self-management (N=13)<br>- I offer more support, education, constant reinforcement, promote self-care, insist on the important of compliance for favourable response to care, and co-manage these patients (N=13)	25 (58)	18 (42)	0	Factors that influence decision; Ease of decision
Beliefs about Capabilities	2	16	1	- I am very confident/somewhat confident in managing neck pain using multimodal treatments (N=16)	16 (100)	0	0	Acceptance, capabilities
Social/Professional Role & Identity	1	21	3	- Managing neck pain patient using multimodal treatment is part of my role as a chiropractor (N=16)<br>- My role is to help patients avoid recurrences and to promote self-care (N=3)<br>- Spinal manipulative Therapy is part of what I do (N=2)	16 (76)	0	5 (24)	Professional role; Professional agreement
Beliefs about Consequences	2	64	5	- Benefits of multimodal- higher treatment include: patient compliance and empowerment, patient education; less medication intake, shorter care, better outcomes (shorter recovery times, decreased pain, decreased headaches, better sleep), and promotes self-care (N=22).<br>- Disadvantages of managing neck pain patients without offering proper advice or prescribing specific exercise	40 (62)	0	24 (38)	Consequence of managing patients with/without multimodal care; Attitudes

				include: poor results (poorer physical functioning, longer recovery time, delayed return to work, high rate of recurrence, increase costs), leading to discouraged patients (depression) and impact on family and discontinuing care) (N=18)<br>- Cervical spine manipulation is generally safe. Advice and exercise are given to patients if SMT is contra-indicated (N=10).<br>-I would not give a patient SMT if it was not to their benefit (N=10)<br>- Not fostering patient dependency to care is important (N=4)				
Intention	1	15	1	-I intend to manage patients using multimodal treatment (N=15)	15 (100)	0	0	Decision to manage patients using multimodal care
Goals	1	21	3	- Education, advice + exercises are equally important (11)/Not as important (2) as delivering SMT (N=13)<br>- Education/advice + exercises are more important in the long term (N=2) - *Important in the early stages as well.*<br>- Empowering and reassuring patients to help them make the best choice is important (N=6)	19 (90)	2 (10)	0	Representations of outcomes/achievement
Emotion	3	3	1	- I get frustrated with non-compliant patients (N=3)	0	3 (100)	0	Own emotions
TOTAL	28	450	39					

a. Statements perceived to increase use of multimodal care (facilitators). b. Statements perceived to reduce use of multimodal care (barriers).
