# Supplementary material for: Fast tracking the design of theory-based KT interventions through a consensus process
Source: Implement Sci. 2015 Feb 11;10:18. doi: 10.1186/s13012-015-0213-5 (PMC4330935; doi:10.1186/s13012-015-0213-5)
Supplement: Additional file 3: — Findings of relevant domains together with illustrative quotes. [file 13012_2015_213_MOESM3_ESM.pdf]

Table 2: Findings of relevant domains together with illustrative quotes.

Domain	Specific beliefs	Selected statements‡
Social Influences	• Seeking advice from colleagues, referring difficult cases to colleagues or seeking a second opinion are important/not important	*"Yes, I believe in co-management with a personal trainer, physiotherapist".* (ON5)
	• Patient reactions influence/don't influence how patients are managed.	*"I would probably leave exercise for a visit or two down the road...when patients are in distress their cognitive functions aren't great and I would leave that information for later on when they can absorb it".* (ON2)
	• Would rather consult guidelines than their peers.	*"...I would tend to refer to the guidelines more than I would refer to my peers".* (NB1)
Environmental Context and Resources	• Lack of important environmental constraints when implementing multimodal care.<br>• Desire for additional exercise equipment or educational resources.<br>• Running behind schedule might affect the use of multimodal treatments on any particular day.	"It would nice to have a website with pictures of stretching, information on counseling skills, a psychologist... to motivate people... Education counselling for me, have it accessible in the office and for patients at home with an app at home....more social consciousness in the media about how treatment with chiropractic has strong research support... Marketing and education to the general public". (ON2)
Reinforcement	• Better clinical outcomes reinforce/don't reinforce the use of multimodal treatments for neck pain patients.	*"Yes, most of the time. But pain is very subjective, ... if there's no improvement with multimodal it would be different".* (ON5)<br>*"Yes I teach them the exercise I'd like them to do. I have them reproduce it in front of me and when they return I ask them if they did it. I ask them again to reproduce it and demonstrate it and then I can modify it. If they*

		haven't done it, it becomes evident. I encourage them to learn it. I show them again and ask them to make up for lost time." (NB1)
Skills	• Ability or proficiency acquired through practice, importance of good doctor-patient communications skills for effectively managing neck pain patients using multimodal care.<br>• Need for counseling skills, and importance of manual and technical skills.	*"Communication is always important with patients. I think it's almost 90% of what we do to explain why we're doing what we're doing. It's important that we explain to them what the treatments are and what they should expect".* (ON4)
Behavioural Regulation	• Managing or changing objectively observed or measured actions.<br>• Regularly monitoring the patient's condition, assessing their motivation to comply with advice and home exercise, encouraging patients to exercise, providing multimodal care, and adapting treatment plans to patients' needs are were considered important strategies for improving patient outcomes.	*"Yes, you reassess every time, checking for movement (ROM), daily function (ADL), monitoring their problems. Outcomes are usually very good. Neck pain can have various causes, and recurrence rate".* (BC1)
Knowledge	• Agreement with the recommendations of the	

	neck pain guidelines<br>• Guidelines are representative of the evidence<br>• Lacking knowledge about exercise	"(I would like) *to refine my knowledge of newer ways to exercise*" (QC1)
	• Confidence in the rigor of the process underlying the development of the guidelines.<br>• Familiarity with the guidelines<br>• Relying only or mostly on their personal experience to inform practice	*"I don't have a written guideline but it's just clinical experience..."* (ON1)
	• Concerned over the definition used for spinal manipulative therapy (SMT) in the guideline.	*"There is no recommendation for how many treatments we might expect for multimodal, manipulation or mobilization...It looked incomplete..."* (QC1)
	• Disappointed by the lack of available research to support the greater use of SMT or to inform specific dosage patterns.	*"[There is] something special about HVLA that I would hope that a guideline would capture that a HVLA can effect...It's a little disappointing."* (ON2)
Memory, Attention and Decision Processes	• Ability to retain information, focusing selectively on aspects of the practice environment, and/or choose between two or more treatment alternatives.<br>• Being challenged by decision making	*"I don't think it's [decision making] is difficult at all because I think that's the way it should be approached by everybody. I have options and I use them as appropriate."* (SK2)<br>*"It'll be easy to enforce the recommendations because I'm already doing it."* (BC2)

	• Practice is in line with guideline recommendations	
	• Relying/not relying on algorithms to make a decision.	*"I don't use an actual rule of thumb. I decide based on clinical presentation. Everyone is unique in their history and presentation so I don't have any specific rule of thumb."* (SK1)
	• Importance of considering patients' psychological factors when deciding whether or not to recommend self-management.	*"Yes I think people who are dealing with yellow flags or psychosocial issues they need a little more hand-holding with exercise and advice. I would bring it up with them on every single visit once I feel they have a handle on it. Constantly reinforcing that they're part of this and part of their recovery."* (ON3)
Social/Professional Role and Identity	• Coherent set of behaviours and personal qualities of chiropractors within their work environment.	"[It is congruent with my role as a chiropractors to employ multimodal care] *Yes. That's what we do. Absolutely."* (ON2)
	• Congruency with the role of the chiropractor to employ multimodal care.	*"Our role is to teach the patient to be self-sufficient and avoid recurrence of the problem."* (QC1)
Belief about Consequences	• Consequence (positive or negative) of action or inaction to the individual in a given situation (i.e., outcomes of a behaviour).<br>• Guideline adherence lead to better outcomes.	*"A higher patient compliance because they're more involved in the process and better educated. Benefits of multimodal would be: shorter recovery times, better outcomes, looking for markers they can identify as recovery, identify increased ROM, decreased pain, decreased headaches, better sleeping patterns."* (NB1)
	• Avoiding creating care dependency.	*"Potentially loss of deterioration of condition, depression, slower return*

	• Failure to adhere to the guidelines would result in negative consequences (slower recovery, decline in patient health).	*to work, effect on earning potential, cost to the economy and employer and effect on the family."* (BC2)
	• SMT considered a safe procedure.<br>• Patients with contra-indications to SMT should receive advice and exercise (self-management).	*"It's extremely safe if you've done the appropriate examination before you consider it as a form of treatment, if not don't.... When in doubt manipulate only when it safe to do so. Use exercise and education."* (SK2)
Beliefs about capabilities	• Sense of acceptance of the truth, reality, or validity about the interviewee's ability, talent, or facility to execute the target behaviours of interest.<br>• Confidence/comfort about managing non-specific neck pain using multimodal treatment, including for acute and chronic neck pain patients.	*"I'm very confident in it. I've had good clinical results myself so there's no reason to question it at this point."* (SK1)
	• Confidence about managing neck pain using multimodal care.	*"I feel for knowing now that what I've seen work in the office I'll probably add that to what I tell my patients. Most people seem quite agreeable to the idea of it being multimodal and doing exercises themselves."* (BC2)
Intention	• Conscious decision to perform a behaviour or a resolution to act in a certain way.	*"I will definitely give them what they ask for. People are very appreciative of the fact that we are doing multimodal."* (BC2)
Goals	• Mental representations of outcomes, or end states	*"It's critically important…Well it's the whole idea of active versus*

	that the interviewee wanted to achieve (e.g. recommend more exercise next week).<br>• Relative importance of education, exercise and patient advice compared to SMT.	*passive treatment…it's the importance of their role in doing exercises."* (AB1)<br>"(The importance) *is at least a third each."* (BC2)
Emotion	• Complex reaction pattern involving experiential, behavioural, and physiological elements, by which the participant attempted to deal with a personally significant event.	*"Patient compliance would help me manage. It can be a problem... That's frustrating."* (ON2)

‡ Each quote is identified by Canadian provincial abbreviation to represent the location of participants (ON: Ontario, NB: New Brunswick, BC: British Columbia, QC: Quebec, SK: Saskatchewan, AB: Alberta) and numerically to represent the specific participant interviewed.
