# Supplementary material for: Fast tracking the design of theory-based KT interventions through a consensus process
Source: Implement Sci. 2015 Feb 11;10:18. doi: 10.1186/s13012-015-0213-5 (PMC4330935; doi:10.1186/s13012-015-0213-5)
Supplement: Additional file 4: — Mapping behaviour change techniques on key domains, proposed KT interventions and actions. [file 13012_2015_213_MOESM4_ESM.pdf]

Additional File 4. Mapping behaviour change techniques on key domains, proposed KT interventions and actions

**TDF Domain**<br>*Behaviour change Technique*<br>**intervention mapping** [129]	**Intervention**<br>(brainstorming)	**Actions** (brainstorming)	**Final intervention component**
**Social Influences**<br><br>*Social processes of encouragement, pressure, support; Modeling/ Demonstration of behaviour by others.*	Opinion leaders, Knowledge Broker, Resource centre	Identifying and train opinion leaders (OLs) among Canadian chiropractic specialists and researchers (through a structured survey among leaders in the profession and other OLs) to deliver key messages	√
	Media Campaign	Question and Answer (Q&A) and possibility for front line clinicians to send in questions by e-mails	
	Website	Entice end-users to apply guideline recommendations, concentrate on doctors of chiropractic (DCs)	√
	Social Media	Enabled with a Q&A section on Facebook and LinkedIn	
	Practice Based Research Network (PBRN)	Create and sustain a PBRN with up to 200 DCs across the country by 2015	√
	Demonstration of behaviour by expert clinician/opinion leader	Produce online webinar and video	√
	Persuasive Communication and Information regarding behaviour outcome	Encourage referrals and multidisciplinary patient management where appropriate	√
**Environmental Context and Resources**<br><br>*Environmental changes (i.e., objects to facilitate behaviour).*<br><br>Note: interventions listed under the	Electronic record keeping systems and related electronic devices	Pilot test the implementation of an Electronic Health Record (EHR) within the PBRN to routinely collect clinical and administrative data	
	Standardized electronic or printed materials as tools for facilitating multimodal or multi-practitioner care	Recommend use of standardized clinical forms to assess and manage patients. Where possible, specify treatment dosage protocols, exercise protocols, referrals, etc.	√ (website)

"Memory, Attention and Decision Processes" domain also apply in this domain.	As a general principle, use a multimodal approach, should be contextualized, interactive and targeted.	Produce webinars, PowerPoints (PPTs), videos, workshops, clinical vignettes, etc. to ease adoption of key guideline recommendations – should be available in different formats (hard copy/electronic resources) on a website intended for practitioners.	√
**Skills**<br><br>*Goal/Target specified: behaviour or outcome; Self-monitoring; Monitoring; Reward/Incentives; Graded tasks (start easy); Increasing skills (problem solving; decision making, goal setting); Rehearsal of relevant skills; Modeling, demonstration of behaviour by others; Homework; Perform behaviour in other settings.*	• Self-monitoring<br><br>• Perform behaviour in different settings	-Context: low patient compliance for self-management (advice on physical activity)<br>-Aim: improve doctor-patient communication skills<br>-Strategy: teach clinicians how to use the 'Brief Action Planning (BAP)' to promote self-management for chronic conditions.<br>-Opportunities:<br>o webinar, video, social media<br>o continuing education, workshops	√ (webinar and video on the BAP
**Reinforcement**<br><br>*Persuasive Communication; Reward/Incentives; Graded tasks (start easy); Increasing skills (problem solving; decision making, goal setting); Motivational Interviewing; Social processes of encouragement, pressure, support* ***(Motivation & Goals).***		-Context: significant range of beliefs about evidence-based practice<br>-Aim: to transform the culture of the profession to one that is guided by evidence-based practice<br>-Expected outcomes:<br>o better patient care and patient health outcomes<br>o better unity of the profession<br>o shift the perception of the general public about chiropractic profession in Canada<br>o increase market share and increase referrals from other providers<br>-Action: demonstrate efficiency and effectiveness of evidence-based practice and of multimodal treatment to improve patient health ('better batting average')	√ (webinar and video)

		-Modes of delivery: Website portal; opinion leaders, social media, DVDs on risk management, APPs (iPhone)	
**Behaviour Regulation**<br><br>*Monitoring; Contract; planning/implementation; Prompts/Triggers/Cues; Use of imagery.*	Action Planning (If/Then plan)<br>• *Computer driven*<br>• *E-records*<br>• *Audit practice*<br>• *Webinars*	-Aim: to provide recurrent messages targeted at clinicians<br>-Audit: possibly based on survey response to clinical vignettes<br>-Integrate action planning strategies within webinars, conferences, workshops<br>-Identify measurement tools to assess patients motivation to comply	√ (webinar)
**Knowledge**<br><br>*Information regarding behaviour, outcome.*	Multimodal interventions to increase understanding of EBP and CPGs, and to increase understanding of key recommendations	-Content: 1) Inform DCs of purpose of evidence-based practice AND what guidelines are for *"to inform decision making along with clinician experience and patients principles and values";* 2) Raise guideline awareness/knowledge regarding multimodal care for neck pain patients<br>-Mode of delivery (paper-base/electronic resources): PPTs, DVDs, webinars, videos, clinical vignettes along with feedback/reinforcement, workshops, social media, PBRN, chiropractic networks/working groups (in a handful of regions, e.g. Regina, McMaster University), publish articles in professional journals/magazines (e.g. Dynamic chiropractic, Canadian Chiropractor)<br>-Opportunity: taking advantage of existing continuing education activities (national/provincial/local society meetings)	√ (webinars, clinical vignettes)
**Memory, Attention and Decision Processes**<br><br>*Self-monitoring;*	• Self-monitoring plan<br>• Audits from existing	1) Standardized office recordkeeping forms:<br>-provide clinicians with and easy-to-use documentation/recordkeeping form (paper and electronic) for initial and follow-up visits: history and exam findings,	√ (forms, checklist, educational handouts,

planning/implementation; Prompts/Triggers/Cues; Motivational Interviewing.<br><br>Note: interventions listed under this domain likely also apply to the domain of Environmental Context and Resources.	record keeping<br>• Automated time management techniques<br>• Facilitate e-communication (smart phones, APPs)<br>• Promote use of patient decision aids<br>• Increase coordination of information across the profession (in parallels with environmental context interventions)	diagnoses, treatments<br>-develop check list with evidence-based options for multimodal treatment with recommended dosage when available<br>- Patient health outcomes submitted electronically (e.g., Care-Response)<br>2) Pre-programmed tablet PCs for use by <u>patients</u> in office waiting rooms:<br>-To view educational articles, videos, podcasts, etc. about evidence-based chiropractic treatment<br>- Surveys about perceived quality of care received (to be complete after care)<br>3) Pre-programmed tablet PCs for use by <u>clinicians</u> in office treatment rooms**:**<br>-To generate ready-made educational handouts, exercise prescriptions, notes and referrals to other health care providers<br>4) Social media and other electronic communication tools/applications:<br>- to facilitate patient education and communication<br>- to disseminate clinical alerts and other evidence-based educational information to patients, which in turn enhances the practitioner's attention to evidence-based patient care pathways<br>5) Content-standardized, but office/practitioner customizable media releases about key evidence-based chiropractic care issues	exercice prescription)

	Prompts, triggers and cues:<br><br>1) Outreach visit<br><br>2) Educational Meetings<br><br>3) Reminders	1) Outreach visit by opinion leader:<br>- Interaction aimed at promoting evidence-based practice for a set of target conditions or clinician behaviour (motivational interviewing seek to incorporate evidence with clinician judgment and patient preference into the application of emerging evidence *(Chilvers 2002)*<br>2) Educational meetings:<br>- Maintain an up-to-date list of CE quality activities within respective jurisdictions<br>- Regularly scheduled interactive rather than didactic meetings with practitioners<br>- Consider types of incentive (e.g., CE credit hours)<br>3) Reminders:<br>- printed cues on promotional 'gifts' with high visibility: widely distributed patient education aids (spine models, educational posters) and commonly utilized office props (diagnostic kit components such as reflex hammers and measuring tapes, and promotional orthopedic supplies (demonstration model back rests, cervical pillows, etc.)<br>- sponsors from health product manufacturers could be solicited to prepare and donate these specially printed produces to practitioners	
	Standardized referral letters	Promote use of standardized referral letters (templates)	
**Social Role and Identity**<br><br>*Social process of encouragement, pressure, support.*	Change agents (opinion leaders)	- Identify and train 1-3 opinion leaders/champions in each jurisdiction (urban and rural) to deliver key messages and to entice a feeling of "ownership" on the guideline content	√
**Belief about Consequences**<br><br>*Persuasive Communication; Information regarding behaviour outcome; Feedback; Self-*	Persuasive Communication; Feedback	1) Create webinars to inform DCs of purpose of evidence-based practice AND what guidelines are for *"to inform decision making along with clinician experience and patients principles and values"*<br>2) Messaging accompanying the guideline (bundled together)	√ (webinar, clinical vignettes

monitoring.<br><br>Note: Beliefs about consequences linked to domains of knowledge and reinforcement.		*"Using guidelines improves patient health"*<br>3) Spin on website of the Ontario Chiropractic Association: it is a way of enhancing practice *"Better outcomes leads to increase referrals"*<br>4) Tie in the environmental context *"using multimodal care won't slow down your practice"*<br>5) Case Study/Clinical vignettes (doctor/patient interaction showing how to provide multimodal care)<br>6) Mass media campaign: inform patients they should expect to receive high quality care	
	Self-monitoring techniques<br><br>1) Standardized office record keeping forms to encourage evidence-based multimodal treatment<br>2) Standardized record keeping audits	1) Standardized office record keeping forms<br>-Agree to a limited set of routinely collected clinical and administrative data to profile clinicians, practice and patients, identify practice patterns & variations and assess impact of dissemination and implementation strategies.<br>-Standardize process across provinces to ease systematic data collection<br><br>2) Clinical audits to promote self-awareness and attention to guidelines-based practice by making practitioners aware of the importance and process of systematic data collection through customary recordkeeping audits	√ (forms)
